# Impact of dexamethasone on postoperative inflammatory markers and recovery outcomes in elderly patients undergoing open lumbar surgery

**DOI:** 10.1016/j.isci.2025.114133

**Published:** 2025-11-19

**Authors:** Huiwen Zhang, Simin Liang, Hui Zhang, Fan Yang, Yonghai Zhang, Feng Wang, Yue Wen, Shaoling Ma, Zhaohui Ge, Hanxiang Ma

**Affiliations:** 1 Department of Anaesthesiology and Perioperative Medicine, General Hospital of Ningxia Medical University, Yinchuan, China; 2 Department of Orthopedics, General Hospital of Ningxia Medical University, Yinchuan, China; 3 Department of Anaesthesiology, Yulin Hospital, The First Affiliated Hospital of Xi’an Jiaotong University, Yulin, China

**Keywords:** Surgery, Orthopedics

## Abstract

The perioperative use of dexamethasone offers anti-inflammatory benefits, but its effects in elderly patients undergoing open lumbar surgery are unclear. In this randomized, double-blind study, individuals aged 65 years and older received a preoperative intravenous dose of dexamethasone (0.15 mg kg^−1^) or placebo. Among 120 enrolled participants, 107 completed all study assessments. On postoperative day 1, plasma C-reactive protein levels were significantly lower with dexamethasone than with placebo (median 10.9 vs. 18.2 mg/L; *p* < 0.001; median difference 7.1 mg/L, 95% CI 3.5–12.4). Wound drainage was also reduced (median 80 vs. 132.5 mL; *p* < 0.001; median difference 52.5 mL, 95% CI 32.5–80.0). Patients receiving dexamethasone showed lower early pain scores, improved recovery, and attenuated inflammatory markers. Transient postoperative hyperglycemia occurred without an increase in infections. These findings support a single preoperative dose of dexamethasone mitigated early inflammation and enhanced recovery in older adults after lumbar spine surgery.

## Highlights

• Preoperative dexamethasone reduced postoperative systemic inflammatory responses
• Lower CRP levels and pro-inflammatory cytokines were observed after lumbar surgery
• Wound drainage volume was significantly reduced in the dexamethasone group
• Improved pain control and recovery quality in elderly surgical patients

## Graphical abstract

**Figure:**
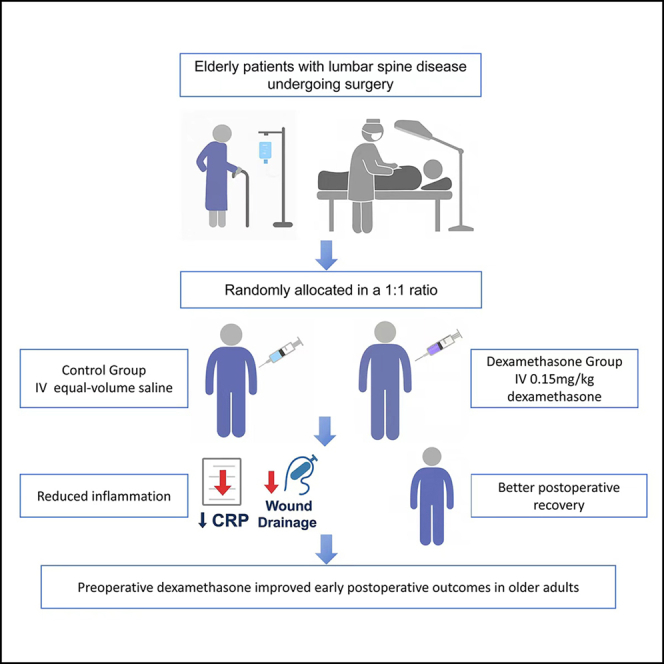

## Introduction

Intense post-operative pain after open lumbar surgery remains a major clinical hurdle and is considered among the three most severe of 179 classified operations.^1^ Within the first 24 h, the majority of patients experience moderate-to-severe discomfort, most likely precipitated by a robust inflammatory reaction to extensive tissue damage.^2^^,^^3^^,^^4^ In elderly patients, whose immunological reserve diminishes over time, this inflammatory escalation may be disastrous, precipitating numerous complications, and markedly impairing recovery and satisfaction.^5^^,^^6^^,^^7^ Accordingly, stringent suppression of inflammation is essential for rapid peri-operative restoration in this population.

Efforts to refine peri-operative care for open lumbar procedures have focused chiefly on analgesic regimens^8^^,^^9^ and technical execution,^10^^,^^11^ with limited investigation into whether tempering the inflammatory cascade in older adults could expedite convalescence.^12^^,^^13^

The immune reaction to surgical injury is complex, altering cellular activity from basal innate defences to adaptive stress pathways.^14^ Inflammation increases capillary permeability, precipitating interstitial oedema, peri-operative fluid overload, and organ dysfunction—sequelae frequently observed after open lumbar procedures with extensive wounds.^15^ Consequently, immunosenescent older adults with multiple comorbidities are particularly vulnerable to inflammation-mediated postoperative complications. Thus far, most investigations have quantified plasma C-reactive protein (CRP) to explore how graded inflammatory responses affect surgical results and infection risk.^16^^,^^17^

Pre-operative prophylactic intravenous glucocorticoids are widely given to improve analgesia and mitigate postoperative nausea and vomiting. Emerging evidence from vascular, orthopedic, and abdominal procedures shows that glucocorticoids blunt systemic inflammation, diminish postoperative pain, and hasten convalescence.^18^^,^^19^^,^^20^ Although previous research has shown the therapeutic advantages of glucocorticoids in orthopedic surgeries, including hip and knee arthroplasty, data focusing on older adults undergoing open lumbar fusion—an operation involving extensive soft tissue disruption and increased susceptibility to inflammation-related complications—are still scarce.

We therefore conducted a randomized, placebo-controlled trial to evaluate intravenous dexamethasone versus saline in modulating postoperative inflammation in this cohort, aiming to refine peri-operative care. We hypothesize that a single pre-operative intravenous dose of dexamethasone (0.15 mg kg^−1^) will reduce postoperative inflammation and drain output compared with saline placebo after open lumbar surgery. This study distinctively integrates systemic inflammatory markers, such as CRP, with local indicators like drain output as co-primary endpoints in an elderly population, thereby filling a spine-specific evidence gap and offering focused guidance for improving perioperative management in this vulnerable group.

## Result

### Study population

**Figure 1:**
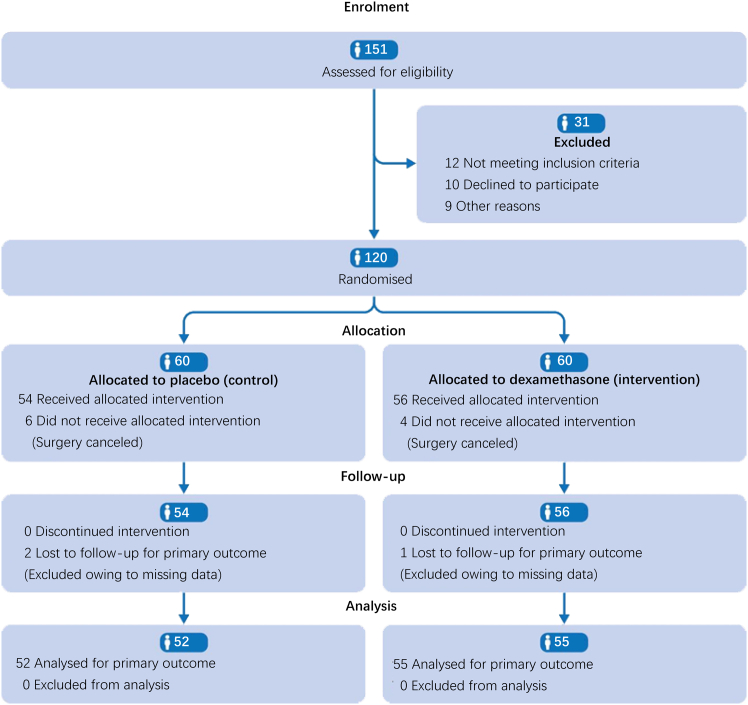
CONSORT flow diagram showing the inclusion and exclusion criteria for patients throughout the study. CONSORT, consolidated standards of reporting trials

The trial was conducted between 15 August 2021 and 1 June 2023. During this interval, 151 candidates were screened; 139 satisfied the inclusion criteria, but 10 declined enrollment and 9 had surgery postponed because of the COVID-19 pandemic, leaving 120 participants who were randomized (control, *n* = 60; dexamethasone, *n* = 60). Subsequently, 10 participants were excluded owing to last-minute surgical cancellations (control, *n* = 6; dexamethasone, *n* = 4). An additional three individuals—two in the control group and one in the dexamethasone group—were removed from the primary analysis because postoperative blood samples were unavailable. Accordingly, the primary-endpoint analysis comprised 52 control and 55 dexamethasone recipients (Figure 1). Patient characteristics and operative data were comparable between the two groups (Table 1).

**Table 1:** Baseline characteristics between the 2 cohorts

Characteristics	Control group (*n* = 52)	dexamethasone group (*n* = 55)
Age (year), median (IQR)	69 (67–72)	68.0 (66–70)
Female (%)	31 (59.6)	35 (63.6)
Height (cm), median (IQR)	165 (160–170)	164 (159–170)
Weight (kg), median (IQR)	65.5 (60.0–70.0)	66.0 (60.0–73.0)
BMI (kg/m^2^), mean (SD)	24.6 (3.2)	24.8 (2.9)
ASA (II/III/IV), No. (%)	20/30/2	21/32/2
Hypertension, No. (%)	19 (36.5)	21 (38.2)
Diabetes^a^, No. (%)	12 (23.1)	16 (29.1)
Preoperative antiplatelet agent, No. (%)	8 (15.4)	9 (16.4)
Hlycated hemoglobin levels (%), mean (SD)	7.2 (1.5)	7.0 (1.3)
Duration of surgery (min), mean (SD)	143.7 (32.5)	133.2 (26.3)
Duration of anesthesia (min), mean (SD)	228.4 (47.0)	213.8 (46.8)
Surgical levels, median (IQR)	3 (2–3)	3 (2–3)
Intraoperative blood loss (mL), median (IQR)	200 (200–400)	200 (200–400)
Intraoperative blood transfusion, No. (%)	6 (11.5)	9 (16.4)
Intraoperative crystalloid volume, median (IQR)	1000 (500–1000)	1000 (500–1000)
Intraoperative colloid volume, median (IQR)	500 (0–500)	500 (0–500)
Intraoperative urine output, median (IQR)	300 (200–400)	400 (200–400)
Hemoglobin concentration at hospital discharge (g/dL), mean (SD)	9.8 (2.1)	10.2 (2.3)

Data are expressed as mean (SD) or median (IQR). Categorical variables were present as No. (%).

BMI, body mass index; ASA, American Society of Anesthesiologists; IQR, interquartile range; SD, standard deviation.

a Diabetes was diagnosed based on preoperative HbA1c ≥ 6.5%, fasting plasma glucose ≥126 mg/dL, or documented medical history with ongoing treatment.

## Primary outcomes

**Figure 2:**
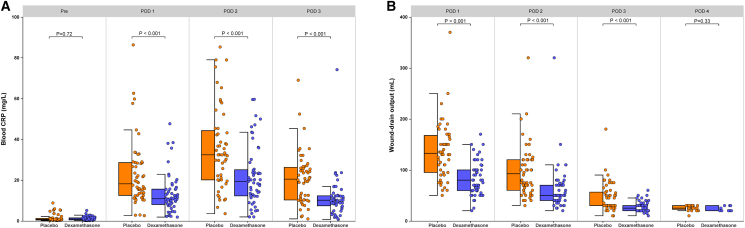
Blood C-reactive protein levels and daily wound-drain output during the perioperative interval (A) Blood CRP during the perioperative interval. (B) Wound drainage output during the perioperative interval. Present the data as a combined box-and-whisker plot with an adjacent scatterplot. In the boxplot on the left, the box depicts the interquartile range, the horizontal line marks the median, and the whiskers denote the minimum and maximum values. In the scatterplot on the right, each circle indicates the data-point distribution density. The Mann-Whitney U test was used. CRP, C-reactive protein; POD, postoperative day.

One hundred and seven patients were included in the co-primary-endpoint analysis. On POD 1, median plasma CRP in the dexamethasone arm was 10.9 mg L^−1^ (interquartile range [IQR] 8.0–15.6) versus 18.2 mg L^−1^ (IQR 12.5–28.6) in controls (*p* < 0.001; median difference 7.1 mg L^−1^, 95% confidence intervals (CI) 3.5–12.4; Figure 2A, Table 2). Using an α = 0.025, dexamethasone also reduced POD 1 drain output to a median of 80 mL (IQR 59.5–100.0) compared with 132.5 mL (IQR 95.0–166.3) in the placebo arm (*p* < 0.001; median difference 52.5 mL, 95% CI 32.5–80.0; Figure 2B, Table 3).

**Table 2:** Blood C-reactive protein levels during the perioperative interval

Time	Control group (*n* = 52)	Dexamethasone group (*n* = 55)	Median difference (95% CI)
**Pre operative (mg/L)**, Median (IQR) [range]	0.74 (0.46–1.22) [0.12–8.80]	0.92 (0.49–1.48) [0.14–5.12]	−0.18 (−0.57 to 0.10)
**POD 1 (mg/L)**, Median (IQR) [range]	18.18 (12.53–28.56) [2.57–86.32]	10.88 (8.00–15.57) [1.85–47.64]	7.08 (3.47–12.35)
**POD 2 (mg/L)**, Median (IQR) [range]	32.40 (20.13–43.73) [3.54–85.29]	19.27 (12.27–25.09) [1.95–59.63]	13.27 (8.55–18.88)
**POD 3 (mg/L)**, Median (IQR) [range]	20.48 (10.31–26.08) [0.97–69.01]	10.15 (7.40–12.30) [0.72–74.16]	10.61 (3.40–14.53)

Data are expressed as median (IQR) [range].

POD, postoperative day; IQR, interquartile range.

**Table 3:** Daily and total wound-drain output volume from postoperative day 1 to day 4

Time	Control group (*n* = 52)	Dexamethasone group (*n* = 55)	Median difference (95% CI)
**POD 1 (mL)**, Median (IQR) [range]	***n* = 52** 132.5 (95.0–166.3) [50–370]	***n* = 55** 80.0 (59.5–100.0) [20–170]	52.5 (32.5–80.0)
**POD 2 (mL)**, Median (IQR) [range]	***n* =52** 92.5 (60.0–120.0) [30–320]	***n* =55** 50.0 (40.0–70.0) [20–320]	13.27 (8.55–18.88)
**POD 3 (mL)**, Median (IQR) [range]	***n* =50** 30.0 (30.0–56.5) [10–180]	***n* =51** 25.0 (20.0–30.0) [10–60]	10.61 (3.40–14.53)
**POD 4 (mL)**, Median (IQR) [range]	***n* =24** 25.0 (23.8–30.0) [10.0–30.0]	***n* =9** 20.0 (20.0–30.0) [ 20.0–30.0]	5.0 (−5.0 to 10.0)
**POD total (mL)**, Median (IQR) [range]	***n* =52** 285.0 (224.0–351.0) [80.0–570.0]	***n* =55** 170.0 (120.0–210.0) [70.0–535.0]	115.0 (60.0–180.0)

Data are expressed as median (IQR) [range]. POD, postoperative day; IQR, interquartile range.

### Secondary outcomes

Relative to the dexamethasone arm, placebo recipients displayed persistently higher plasma CRP levels and larger daily drain outputs on postoperative days 2–3 (Figures 2A and 2B). On POD 3, median CRP was 20.5 mg L^−1^ (IQR 10.3–26.1) with placebo versus 10.2 mg L^−1^ (IQR 7.4–12.3) with dexamethasone (*p* < 0.001; Table 2); corresponding median drain volumes were 30 mL (IQR 30–56.5) and 25 mL (IQR 20–30), respectively (*p* < 0.001; Table 3). Cumulative drainage was reduced in the dexamethasone group: median 170 mL (IQR 120–210) compared with 285 mL (IQR 224–351) in controls (*p* < 0.001; median difference 115 mL, 95% CI 60–180; Table 3). Mean drain duration was also shorter after dexamethasone—4.1 (1.1) days versus 5.4 (1.2) days with placebo (*p* = 0.003; Table 4).

**Table 4:** Secondary outcomes

Variable	Control group (*n* = 52)	Dexamethasone group (*n* = 55)	RR or difference (95% CI)^a^	*p* value^b^
Time to extubation, mean (SD), min	39.9 (20.3)	42.5 (25.6)	−2.61 (−10.39 to 8.71)	0.643
Duration in PACU, mean (SD), min	80.3 (20.4)	85.1 (23.8)	−4.83 (−25.66 to 20.32)	0.513
ICU admission rate, No. (%)	5 (9.6)	7 (12.7)	1.32 (0.45–3.91)	0.761
PONV, No. (%)				
0–24 h	23 (44.2)	10 (18.2)	0.41 (0.22–0.78)	0.006
24–36 h	14 (26.9)	6 (10.9)	0.41 (0.17–0.97)	0.047
36–48 h	7 (13.5)	4 (7.3)	0.54 (0.17–1.74)	0.351
Rescue analgesia, No. (%)				
0–12 h	21 (40.4)	8 (14.5)	0.36 (0.18–0.74)	0.004
12–24 h	25 (48.1)	13 (23.6)	0.49 (0.28–0.85)	0.009
24–36 h	19 (36.5)	9 (16.4)	0.45 (0.22–0.90)	0.027
36–48 h	13 (25.0)	8 (14.5)	0.58 (0.26–1.29)	0.225
Postoperative complication, No. (%)	9 (17.3)	13 (23.6)	1.37 (0.64–2.63)	0.462
Surgical site infection	1 (1.9)	2 (3.6)	−1.69 (−10.23 to 6.50)	NA
Nerve injury	2 (3.8)	3 (5.5)	−1.63 (−11.11 to 7.72)	NA
Pulmonary embolism	1 (1.9)	0 (0)	1.82 (−4.67 to 9.61)	NA
Cerebrospinal fluid leak	1 (1.9)	2 (3.6)	−1.69 (−10.23 to 6.50)	NA
Delirium	4 (7.7)	6 (10.9)	−3.25(-14.73 to 8.20)	NA
Cerebrospinal fluid leaks	0 (0)	0 (0)	0 (−6.31 to 6.53)	NA
Length of hospital stay, mean (SD), day	9.8 (2.0)	10.0 (2.4)	0.29 (−0.56 to 1.15)	0.503
Duration of drain indwelling, mean (SD), day	5.4 (1.2)	4.1 (1.1)	−1.32 (−2.19 to −0.45)	0.003
Readmission before day 30, No. (%)	2 (3.9)	5 (9.1)	2.36 (0.48–11.65)	0.439
Patient satisfaction, No. (%)				
Very satisfied	41 (78.8)	40 (72.7)	6.12 (−10.18 to 21.85)	0.788
Satisfied	7 (13.5)	10 (18.2)	−4.72 (−18.64 to 9.54)	
Unsatisfied	3 (5.8)	3 (5.5)	0.31 (−9.82 to 10.82)	
Very unsatisfied	1 (1.9)	2 (3.6)	−1.71 (−10.54 to 6.9)	
Antalgic consumption at day 30, No. (%)	17 (32.7)	24 (43.6)	1.34 (0.83–2.14)	0.253

Data are expressed as mean (SD). PACU, post-anesthesia care unit; PONV, postoperative nausea and vomiting; ICU, intensive care unit; RR, risk ratio.

a RRs were reported for categorical outcomes, whereas mean differences were presented for continuous data.

b Fisher exact test, χ2 test, and *t* test were used as appropriate.

**Figure 3:**
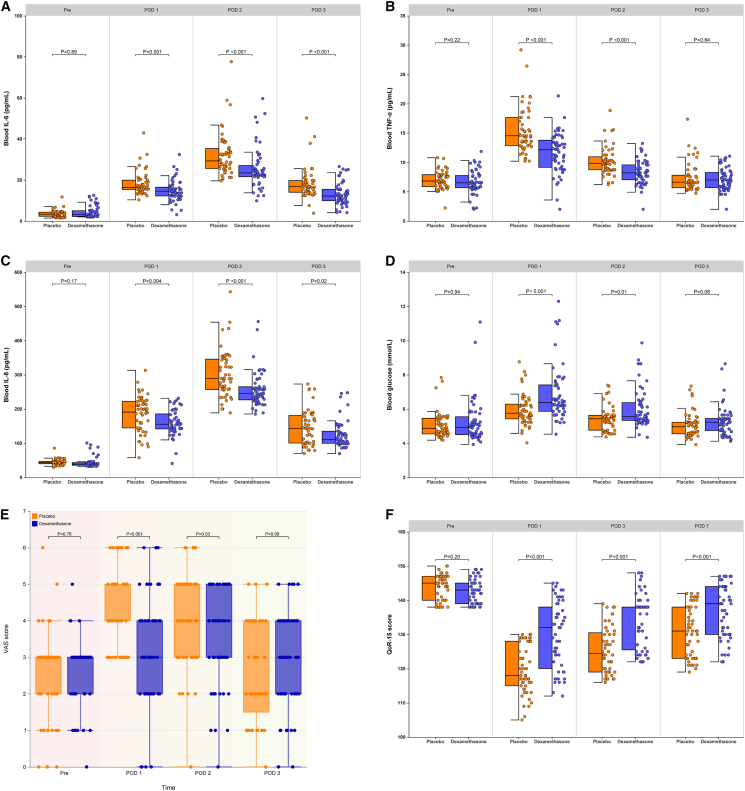
IL-6, TNF-α, IL-8, blood glucose, VAS score, and QoR-15 score (A) Blood IL-6 during the perioperative interval. (B) Blood TNF-α during the perioperative interval. (C) Blood IL-8 during the perioperative interval. (D) Blood glucose during the perioperative interval. (E) Pain VAS score (0–10 scale, with 0 meaning “no pain” and 10 meaning “the worst pain imaginable”). (F) QoR-15 score during the perioperative interval. Present the data as a combined box-and-whisker plot with an adjacent scatterplot. In the boxplot on the left, the box depicts the interquartile range, the horizontal line marks the median, and the whiskers denote the minimum and maximum values. In the scatterplot on the right, each circle indicates the data-point distribution density. The Mann-Whitney U test was used. IL, interleukin; TNF, tumor necrosis factor; VAS, visual analog scale; QoR, quality of recovery. POD, postoperative day.

Relative to the placebo arm, dexamethasone recipients exhibited lower plasma concentrations of CRP, interleukin-6, interleukin-8, and tumor necrosis factor-α on postoperative days 2–3 (Figures 3A–3C; Tables 1 and S1–S3). Postoperative leukocyte counts and neutrophil fractions did not differ significantly between groups (Figures S1 and S2, Tables S4 and S5). Among patients with drains left *in situ*, daily outputs on postoperative days 2–3 were consistently smaller with dexamethasone than with placebo (Table 3). Cumulative drainage was likewise reduced in the dexamethasone cohort: median 170 mL (IQR 120–210) versus 285 mL (IQR 224–351) in controls (*p* < 0.001; median difference 115 mL, 95% CI 60–180; Table 3).

Relative to placebo, patients receiving dexamethasone exhibited higher median postoperative blood-glucose concentrations, persisting on day 1 (*p* < 0.001) and day 2 (*p* = 0.01), but returning to parity by day 3 (Figure 3D, Table S6). No episodes of diabetic ketoacidosis occurred in either study arm. In a post-hoc subgroup analysis, no significant differences were observed in hyperglycemia duration or severity between diabetic (*n* = 28) and non-diabetic (*n* = 79) patients (*p* = 0.32 for peak glucose; median difference 12 mg/dL, 95% CI -8 to 32).

Relative to the placebo arm, dexamethasone recipients reported lower movement-evoked visual-analogue pain scores across the first three postoperative days (Figure 3E, Table S7). Kaplan-Meier analysis of time to first rescue analgesia appears in Figure S3; a log rank test confirmed a longer analgesia-free interval in the dexamethasone arm versus placebo (*p* = 0.001). Additionally, a smaller proportion of dexamethasone recipients required rescue analgesia during the 0–12 h, 12–24 h, and 24–36 h postoperative intervals (Table 4); no inter-group difference was observed in the 36–48 h period. Patients who received dexamethasone also exhibited higher QoR-15 scores on postoperative days 1, 3, and 7 than those given placebo (Figure 3F, Table S8).

Within the first 36 h, dexamethasone recipients experienced fewer episodes of postoperative nausea and vomiting, with no inter-group difference thereafter (Table 4). Notably, drain removal occurred sooner in dexamethasone recipients, averaging 4.1 (1.1) days compared with 5.4 (1.2) days in the placebo arm (*p* = 0.003; mean difference −1.32 days, 95% CI –2.19 to −0.45; Table 4). Twenty-two serious postoperative complication, unrelated to the study medication, were documented (Table 4). The aggregate incidence of serious events did not differ between groups (*p* = 0.46; RR 1.37, 95% CI 0.64–2.63), encompassing surgical-site infection (1.9% vs. 3.6%), nerve injury (3.8% vs. 5.5%), pulmonary embolism (1.9% vs. 0%), cerebrospinal fluid leak (1.9% vs. 3.6%), delirium (7.7% vs. 10.9%), and cerebrospinal fluid leaks (0% vs. 0%) for dexamethasone and placebo, respectively. Reasons for 30-day readmissions were wound dehiscence (*n* = 3), pain (*n* = 3), and urinary infection (*n* = 1), with no group differences. There were no significant differences in non-invasive hemodynamic parameters between the two groups postoperatively (Figure S4).

## Discussion

In this randomized controlled trial, we investigated the impact of a single preoperative intravenous dose of dexamethasone (0.15 mg kg^−1^) on postoperative inflammatory responses in elderly patients undergoing open posterior lumbar interbody fusion. Our findings demonstrate that dexamethasone significantly reduces plasma CRP concentrations and wound-drain output on postoperative day 1, attenuates systemic and local inflammatory markers through day 3, and improves recovery metrics such as pain scores, nausea, and quality of recovery, without increasing infection rates or major complications.

Reductions in plasma concentrations of CRP and pro-inflammatory cytokines (IL-6, IL-8, and TNF-α) imply that dexamethasone attenuates the acute-phase inflammatory cascade elicited by surgical injury. Such attenuation is probably mediated by glucocorticoid-induced suppression of nuclear factor-κB signaling, which decreases cytokine gene transcription and fortifies the vascular endothelium, thereby lowering capillary leak and interstitial fluid formation.^21^^,^^22^ The 52.5 mL median reduction in POD 1 drain output likely reflects decreased local exudation and tissue edema, correlating with shorter drain indwelling time (mean difference −1.32 days, 95% CI −2.19 to −0.45) and potentially facilitating earlier mobilization, though direct measures of swelling or ambulation were not assessed. The transient hyperglycemia observed on postoperative days 1–2 is consistent with glucocorticoid-induced gluconeogenesis and resolved by day 3, suggesting minimal metabolic disruption in this nondiabetic cohort. This finding parallels the PADDAG trial^23^, which documented comparable short-lived glycemic elevations in noncardiac surgical patients without lasting metabolic effects. Improved analgesia and diminished nausea likely reflect anti-inflammatory modulation of nociceptive pathways and the chemoreceptor trigger zone, respectively, thereby contributing to expedited drain removal.

The QoR-15 score differed significantly in favor of the dexamethasone cohort. This advantage may reflect enhanced analgesia and attenuated inflammation—which jointly lessen fatigue and improve physical comfort and emotional state—while glucocorticoid-related psychotropic effects could further bolster perceived well-being.

Our findings corroborate recent literature on peri-operative glucocorticoids in orthopedic surgery and, importantly, broaden the evidence base to encompass a frail geriatric cohort. Consistent with a review demonstrating a 30%–50% CRP reduction after total knee arthroplasty^24^, we observed a 40% median decline in POD-1 CRP, underscoring similar anti-inflammatory potency. Likewise, a study of patients undergoing total hip arthroplasty found reduced IL-6 with low-dose dexamethasone^25^, echoing our cytokine pattern yet in a different operative setting; by targeting lumbar fusion we close a spine-specific evidence gap. Drain-output findings diverge: colorectal-surgery data showed no benefit,^26^ whereas our 52.5 mL median reduction likely reflects the larger soft-tissue dissection intrinsic to lumbar surgery, underscoring procedure-specific physiology. Methodological strength stems from combining systemic (CRP) and local (drain volume) metrics as co-primary endpoints, in contrast with earlier trials that focused solely on analgesia.^27^^,^^28^ Notably, leukocyte and neutrophil counts were comparable between groups, contradicting the assumption that dexamethasone-induced immunosuppression would diminish their proliferation. Postoperative leukocyte kinetics is governed by a multifactorial physiological milieu. A plausible explanation is the routine administration of postoperative antibiotics in both cohorts; leukocytes and neutrophils respond robustly to antimicrobial therapy, whereas circulating cytokines remain comparatively unaffected.

Our choice of dexamethasone 0.15 mg kg^−1^, regarded as a low dose, concurs with emerging evidence that lower peri-operative doses optimize the efficacy-safety balance.^29^^,^^30^^,^^31^ Systematic reviews demonstrate that 0.05–0.1 mg kg^−1^ (or 8–10 mg fixed) regimens lower pain, opioid need, and inflammatory indices without increasing infection or impaired healing^32^; our reductions in CRP, cytokines, and drainage mirror these findings. Conversely, contemporary evidence indicates that high-dose dexamethasone confers no distinct benefit for patients displaying hyper- or hypoalgesia, nor for certain operations—such as cardiac surgery—and may instead provoke persistent postoperative dysglycaemia, impede wound repair, and increase infection risk. Two clinical trials, for example, investigated a 1 mg kg^−1^ dexamethasone regimen in total-knee-arthroplasty patients stratified by pain sensitivity. In participants with pronounced nociceptive responsiveness, the regimen reduced 24-h moderate-to-severe pain and enhanced analgesic recovery without meaningful adverse effects, whereas no benefit emerged in low-pain responders.^33^^,^^34^ Conversely, administering the identical dose to hip-arthroplasty candidates predicted to experience severe pain did not improve postoperative discomfort or functional recovery.^35^ Collectively, potential ceiling effects of dexamethasone and its dose-dependent hazards—especially in insulin-dependent diabetic patients—should be critically appraised. The 0.15 mg kg^−1^ dose administered in this study exceeded the standard recommendation by a small margin. This dosage choice was partially informed by findings from our preliminary investigation. Moreover, this dosage corresponds to the moderate-to-low range reported in contemporary clinical protocols.^36^^,^^37^

These results deepen current insight into glucocorticoid regulation of age-related immunoinflammation and have direct peri-operative relevance for geriatric spine surgery. Because dexamethasone attenuates inflammation without raising infection rates, it could be incorporated into standard protocols to hasten recovery, decrease healthcare costs, and enhance patient satisfaction, thereby informing guidance issued by organizations such as the American Society of Anesthesiologists. Future research may examine multimodal regimens that pair dexamethasone with targeted anti-cytokine agents, thereby shaping policy for surgical care in the elderly.

In summary, preoperative dexamethasone attenuates postoperative inflammation and enhances recovery in elderly lumbar surgery patients, offering a safe, low-cost intervention with broad clinical potential. Prospective research should validate these benefits in multicenter cohorts and explore dose-response relationships to optimize geriatric perioperative care.

### Limitations of the study

The single-center design and relatively homogeneous Chinese elderly population may limit applicability to diverse demographics or centers with varying protocols (e.g., routine tranexamic acid use or minimally invasive techniques). Multicenter trials are needed to confirm broader relevance. The pilot study informing sample size calculations involved only 14 participants, potentially leading to unreliable effect size estimates and risk of under- or over-powering; however, our achieved power for primary endpoints remained robust at >90%. Additionally, we focused on standard inflammatory markers (CRP, IL-6, IL-8, and TNF-α); future studies could include advanced mediators such as leukotriene B4 and 20-hydroxyeicosatetraenoic acid^38^ to further elucidate resolution pathways. Patient-centered outcomes beyond QoR-15, such as chronic pain, time to mobilization, and long-term functional status (e.g., via the oswestry disability index), were not assessed, limiting insights into physical recovery. The per-protocol approach, necessitated by missing data, may overestimate treatment efficacy. Future investigations should employ multicentre designs and integrate molecular biomarkers, such as transcriptomic sequencing, to elucidate underlying mechanisms.

## Resource availability

### Lead contact

Further information and requests should be directed to the lead contact, Prof. Hanxiang Ma (mahanxiang@hotmail.com).

### Materials availability

This study did not generate new unique reagents.

### Data and code availability

The data for this article will be shared at a reasonable request by the corresponding author. This paper does not report any original code. Any additional information require to reanalyze the data reported in this paper is available from the lead contact upon request.

## Acknowledgments

We gratefully acknowledge the participants and staff members at the General Hospital of Ningxia Medical University. We are also deeply thankful to the Ningxia Science and Technology Department and 10.13039/501100004179Ningxia medical university for their generous financial support. The Ningxia Key Research and Development Program (2021BEG03048 and 2023BEG03013), and a University-level research grant from Ningxia Medical University (no. XZ2023030).

## Author contributions

Writing – original draft, project administration, methodology, investigation, formal analysis, data curation, Huiwen Zhang; writing – original draft, methodology, investigation, formal analysis, data curation, S.L. investigation, data curation, Hui Zhang; methodology, formal analysis, F.Y.; data curation, Y.Z.; data curation, F.W.; data curation, Y.W.; methodology, S.M.; co-corresponding author; writing – review & editing, supervision, project administration, conceptualization, Z.G.; primary corresponding author, writing – review & editing, supervision, project administration, methodology, conceptualization, H.M.

## Declaration of interests

The authors declare no conflicts of interest.

## STAR★Methods

### Key resources table

**Table:** 

REAGENT or RESOURCE	SOURCE	IDENTIFIER
**Software and algorithms**		

PASS	NCSS	https://www.ncss.com/software/pass
SPSS Statistics version 27	IBM	https://www.ibm.com/analytics/pass-statistics-software
GraphPad Prism, version 7.0	GraphPad Software	https://www.graphpad.com/

**Other**		

Ethics Committee approval (China)	N/A	Ningxia Medical University General Hospital Ethics Committee (Approval No. 2020-04)
Trial registration	N/A	NCT04981093

### Experimental model and study participant details

This randomized, double-blind clinical trial enrolled patients aged 65 years and older with American Society of Anesthesiologists (ASA) physical status II–IV undergoing elective open posterior lumbar decompression and fusion surgery at the General Hospital of Ningxia Medical University (Yinchuan, China). Research nurses recruited participants during routine preoperative anesthesia assessments. Exclusion criteria included poorly controlled diabetes (HbA1c > 9.0%), current or recent systemic infection, chronic glucocorticoid use, prior lumbar surgery, anticipated minor procedures with incision <5 cm, and delayed extubation beyond 2 h. Full eligibility criteria are listed in the trial protocol.

The study was conducted at a tertiary academic center and approved by the Ningxia Medical University General Hospital Ethics Committee (Approval No. 2020-04; approval date: August 17, 2020). Written informed consent was obtained from all participants prior to enrollment. The trial was registered on ClinicalTrials.gov before recruitment (NCT04981093). The approved protocol version and trial documents are available upon request from the corresponding author.

### Method details

#### Randomization and blinding

Participants who consented to join the study and met eligibility criteria were randomly allocated to receive either intravenous dexamethasone (0.15 mg/kg) or placebo following the induction of general anesthesia. Randomization was performed using computer-generated sequences in blocks of 10. To ensure allocation concealment, sequentially numbered and opaque envelopes were utilized, which were sealed. An anesthesia resident, who had no involvement in patient care or outcome assessment, opened each envelope. Based on the allocation, a nurse, also not engaged in patient care, prepared a 5 mL syringe that either contained dexamethasone (0.15 mg kg^−1^) diluted in saline or a placebo of 0.9% saline. Treatment assignments remained masked to patients, clinical teams, and investigators until data analysis was complete.

#### Anesthesia and study interventions

No sedative premedication was administered. On arrival in the preoperative holding area, an IV line was established, and standard monitoring was initiated. General anesthesia was induced with midazolam (0.03 mg/kg), sufentanil (0.3–0.4 μg/kg), etomidate (0.3 mg/kg), and rocuronium (0.8 mg/kg). After securing the airway, anesthesia was maintained using sevoflurane (0.7–1.0 MAC) alongside continuous infusions of propofol (4–12 mg/kg/h) and remifentanil (0.1–1.0 μg/kg/min). Additional rocuronium was given as needed to sustain neuromuscular blockade. Intraoperative hemodynamics were controlled within 20% of pre-induction values. All procedures were conducted by a consistent orthopedic surgical team using a standardized posterior approach. Upon completion, a closed-suction drain was placed, and 20 mL of 0.25% ropivacaine was infiltrated around the wound.

Patients were transferred to the PACU for routine monitoring. Each received intravenous ondansetron (4 mg) to reduce the risk of postoperative nausea and vomiting. PACU nurses, unaware of group assignments, oversaw recovery until discharge criteria were satisfied. Patients were then moved to the general ward. Postoperative analgesia included patient-controlled intravenous analgesia (PCIA) using sufentanil (100 μg in 100 mL saline; 2 mL bolus; 15-min lockout; no basal infusion), intravenous parecoxib (40 mg every 12 h), and oral paracetamol (650–1000 mg every 6 h). The acute pain service reviewed patients twice daily and adjusted analgesia as needed.

#### Outcomes

Primary outcomes were C-reactive protein (CRP) levels and wound drain output measured on postoperative day 1. Blood sampling and drain measurements were standardized and recorded at consistent morning time points. Secondary endpoints included inflammatory markers (IL-6, IL-8, TNF-α) on postoperative days 2–3; cumulative drain volume; pain scores (VAS on POD 1–3); capillary glucose trends over 72 h; QoR-15 scores at baseline and on POD 1, 3, and 7; ICU admission; hospital stay duration; and a range of postoperative complications. Drain patency was checked daily; drains were removed after POD 2 if output remained below 30 mL. Any empty bags on POD 1–2 were verified for obstruction or exclusion. A follow-up phone interview was conducted one week post-discharge to assess late complications. All outcome assessments were performed by personnel blinded to treatment assignment.

### Quantification and statistical analysis

#### Sample size calculation

To evaluate early postoperative inflammatory response, plasma C-reactive protein (CRP) concentration and wound-drain output on postoperative day 1 were treated as co-primary endpoints. Several secondary measures were recorded, including inflammatory cytokine levels (IL-6, IL-8, TNF-α) on postoperative days 2 and 3, total drain output over the first 72 h, pain intensity assessed by VAS on postoperative days 1 through 3, capillary glucose values during the same period, recovery quality measured using the QoR-15 at four timepoints (preoperative baseline, POD 1, 3, and 7), along with ICU admission, length of stay, and postoperative complications.

Sample size was estimated based on pilot data from 14 older patients who had undergone open lumbar surgery, where CRP levels on postoperative day 1 averaged 8.3 mg/L (SD 3.8) in the dexamethasone group and 12.4 mg/L (SD 5.6) in controls. Assuming a pooled standard deviation of 5.6 mg/L and setting the two-sided significance level at 0.025 (to account for multiple primary outcomes), we determined that 48 participants per group would yield 90% power to detect a meaningful difference in CRP. This same sample size also allowed for >90% power to detect a standardized 64% difference in drain output. Factoring in potential attrition, we aimed to enroll 120 participants (60 per group).

#### Statistical details of experiments

Across the Results, the flow diagram, and Tables 1, 2, 3, and 4, the symbol n indicates the participant count. The footnotes of each table (Tables 1, 2, 3, and 4) define the reporting formats for outcomes—mean (SD), median (IQR), No. (%), and median difference with 95% CI.

#### Statistical analysis

All study data were managed in an electronic database and analyzed using Microsoft Office Excel 2010 and IBM SPSS Statistics for Windows, version 20.0 (IBM Corp., Armonk, NY). Descriptive and inferential analyses were conducted for all variables, with results displayed in tables, graphs, and figures. Because some participants lacked primary-endpoint data, analyses followed a per-protocol approach. Post-operative-day-1 CRP concentrations and drain outputs were compared with mixed-effects linear regression, adjusting for treated-segment count, baseline CRP, age, and sex as fixed covariates. Secondary endpoints were analyzed with χ^2^ or Fisher exact tests for categorical data and t- or Mann-Whitney U tests for continuous data; proportions were expressed as risk ratios, and continuous variables as mean differences with 95% confidence intervals. Repeated-measures generalised linear models contrasted pain scores across time points. To control family-wise error for the co-primary endpoints, a two-sided α = 0.025 was applied. All other tests were two-sided with α = 0.05. Analyses were executed in GraphPad Prism 7.0.

### Additional resources

The trial was registered on ClinicalTrials.gov before recruitment (NCT04981093; registration date: August 15, 2021; principal investigator: Hanxiang Ma).

## References

[1] Gerbershagen H.J., Aduckathil S., van Wijck A.J.M., Peelen L.M., Kalkman C.J., Meissner W. (2013). Pain intensity on the first day after surgery: a prospective cohort study comparing 179 surgical procedures. Anesthesiology.

[2] Khor S., Lavallee D., Cizik A.M., Bellabarba C., Chapman J.R., Howe C.R., Lu D., Mohit A.A., Oskouian R.J., Roh J.R. (2018). Development and Validation of a Prediction Model for Pain and Functional Outcomes After Lumbar Spine Surgery. JAMA Surg..

[3] Hong B., Baek S., Kang H., Oh C., Jo Y., Lee S., Park S. (2023). Regional analgesia techniques for lumbar spine surgery: a frequentist network meta-analysis. Int. J. Surg..

[4] Shaygan M., Zamani M., Jaberi A., Eghbal K., Dehghani A. (2023). The impact of physical and psychological pain management training on pain intensity, anxiety and disability in patients undergoing lumbar surgeries. Spine J..

[5] Mannion A.F., Fekete T.F., O'Riordan D., Porchet F., Mutter U.M., Jeszenszky D., Lattig F., Grob D., Kleinstueck F.S. (2013). The assessment of complications after spine surgery: time for a paradigm shift?. Spine J..

[6] Boccardi V., Marano L. (2024). Improving geriatric outcomes through nutritional and immunonutritional strategies: Focus on surgical setting by a comprehensive evidence review. Ageing Res. Rev..

[7] Gaudilliere B., Xue L., Tsai A.S., Gao X., McAllister T.N., Tingle M., Porras G., Feinstein I., Feyaerts D., Verdonk F. (2025). Infusion of young donor plasma components in older patients modifies the immune and inflammatory response to surgical tissue injury: a randomized clinical trial. J. Transl. Med..

[8] Waelkens P., Alsabbagh E., Sauter A., Joshi G.P., Beloeil H., PROSPECT Working group∗∗ of the European Society of Regional Anaesthesia and Pain therapy ESRA (2021). Pain management after complex spine surgery: A systematic review and procedure-specific postoperative pain management recommendations. Eur. J. Anaesthesiol..

[9] Oh S.K., Lim B.G., Won Y.J., Lee D.K., Kim S.S. (2022). Analgesic efficacy of erector spinae plane block in lumbar spine surgery: A systematic review and meta-analysis. J. Clin. Anesth..

[10] Oosterhuis T., Ostelo R.W., van Dongen J.M., Peul W.C., de Boer M.R., Bosmans J.E., Vleggeert-Lankamp C.L., Arts M.P., van Tulder M.W. (2017). Early rehabilitation after lumbar disc surgery is not effective or cost-effective compared to no referral: a randomised trial and economic evaluation. J. Physiother..

[11] Soffin E.M., Beckman J.D., Tseng A., Zhong H., Huang R.C., Urban M., Guheen C.R., Kim H.J., Cammisa F.P., Nejim J.A. (2020). Enhanced Recovery after Lumbar Spine Fusion: A Randomized Controlled Trial to Assess the Quality of Patient Recovery. Anesthesiology.

[12] Demura S., Takahashi K., Murakami H., Fujimaki Y., Kato S., Tsuchiya H. (2013). The influence of steroid administration on systemic response in laminoplasty for cervical myelopathy. Arch. Orthop. Trauma Surg..

[13] Xu C., Wang X., Wang M., Wang D., Kong S., Zhou Q., Lu J., Wang A. (2025). Implication of Preoperative Intravenous Dexamethasone on Pain and Nausea in Late-Stage Elderly Total Knee Arthroplasty Patients: A Randomized Double-Blind Trial. J. Arthroplast..

[14] Bain C.R., Myles P.S., Corcoran T., Dieleman J.M. (2023). Postoperative systemic inflammatory dysregulation and corticosteroids: a narrative review. Anaesthesia.

[15] Funk D.J., HayGlass K.T., Koulack J., Harding G., Boyd A., Brinkman R. (2015). A randomized controlled trial on the effects of goal-directed therapy on the inflammatory response open abdominal aortic aneurysm repair. Crit. Care.

[16] Yang Q., Li M., Cao X., Lu Y., Tian C., Sun M., Lai H., Tian J., Li J., Ge L. (2022). An umbrella review of meta-analyses on diagnostic accuracy of C-reactive protein. Int. J. Surg..

[17] Hoeller S., Roch P.J., Weiser L., Hubert J., Lehmann W., Saul D. (2021). C-reactive protein in spinal surgery: more predictive than prehistoric. Eur. Spine J..

[18] Hannon C.P., Fillingham Y.A., Mason J.B., Sterling R.S., Casambre F.D., Verity T.J., Woznica A., Nelson N., Hamilton W.G., Della Valle C.J. (2022). The Efficacy and Safety of Corticosteroids in Total Joint Arthroplasty: A Direct Meta-Analysis. J. Arthroplast..

[19] Myles P.S., Dieleman J.M., Munting K.E., Forbes A., Martin C.A., Smith J.A., McGiffin D., Verheijen L.P.J., Wallace S., DECS-II Investigators and the ANZCA Clinical Trials Network (2024). Dexamethasone for Cardiac Surgery: A Practice Preference-Randomized Consent Comparative Effectiveness Trial. Anesthesiology.

[20] Cihoric M., Kehlet H., Lauritsen M.L., Højlund J., Kanstrup K., Kärnsund S., Foss N.B. (2024). Preoperative high dose of dexamethasone in emergency laparotomy: randomized clinical trial. Br. J. Surg..

[21] Zargar-Shoshtari K., Sammour T., Kahokehr A., Connolly A.B., Hill A.G. (2009). Randomized clinical trial of the effect of glucocorticoids on peritoneal inflammation and postoperative recovery after colectomy. Br. J. Surg..

[22] Corcoran T.B., Martin C., O'Loughlin E., Ho K., Chan M., Forbes A., Leslie K., Myles P. (2023). Dexamethasone and persistent wound pain: a prespecified analysis of the randomised Perioperative Administration of Dexamethasone and Infection (PADDI) trial. Br. J. Anaesth..

[23] Nielsen R.V., Siegel H., Fomsgaard J.S., Andersen J.D.H., Martusevicius R., Mathiesen O., Dahl J.B. (2015). Preoperative dexamethasone reduces acute but not sustained pain after lumbar disk surgery: a randomized, blinded, placebo-controlled trial. Pain.

[24] Lei Y., Huang Z., Huang Q., Pei F., Huang W. (2020). Is a split-dose intravenous dexamethasone regimen superior to a single high dose in reducing pain and improving function after total hip arthroplasty? A randomized blinded placebo-controlled trial. Bone Joint Lett. J..

[25] Lei Y., Huang Z., Huang Q., Pei F., Huang W. (2022). Dose optimization of intravenous dexamethasone for total knee arthroplasty: when two is not better than one. Arch. Orthop. Trauma Surg..

[26] Lei Y., Huang Z., Huang Q., Huang W., Pei F. (2020). Repeat Doses of Dexamethasone up to 48 Hours Further Reduce Pain and Inflammation After Total Hip Arthroplasty: A Randomized Controlled Trial. J. Arthroplast..

[27] Toner A.J., Ganeshanathan V., Chan M.T., Ho K.M., Corcoran T.B. (2017). Safety of Perioperative Glucocorticoids in Elective Noncardiac Surgery: A Systematic Review and Meta-analysis. Anesthesiology.

[28] Nielsen N.I., Kehlet H., Gromov K., Troelsen A., Husted H., Varnum C., Kjærsgaard-Andersen P., Rasmussen L.E., Pleckaitiene L., Foss N.B. (2023). High-dose dexamethasone in low pain responders undergoing total knee arthroplasty: a randomised double-blind trial. Br. J. Anaesth..

[29] Nielsen N.I., Kehlet H., Gromov K., Troelsen A., Husted H., Varnum C., Kjærsgaard-Andersen P., Rasmussen L.E., Pleckaitiene L., Foss N.B. (2022). High-dose steroids in high pain responders undergoing total knee arthroplasty: a randomised double-blind trial. Br. J. Anaesth..

[30] Nielsen N.I., Kehlet H., Gromov K., Troelsen A., Husted H., Varnum C., Kjærsgaard-Andersen P., Rasmussen L.E., Pleckaitiene L., Foss N.B. (2023). High dose dexamethasone in high pain responders undergoing total hip arthroplasty: A randomized controlled trial. Eur. J. Anaesthesiol..

[31] Chen H., Wang Y., Jiang K., Xu Z., Jiang Y., Wu Z., Lu X., Wang C., Weng Y., Wang W. (2024). The Effect of Perioperative Dexamethasone on Postoperative Complications After Pancreaticoduodenectomy: A Multicenter Randomized Controlled Trial. Ann. Surg..

[32] Bouras M., Clément A., Schirr-Bonnans S., Mauduit N., Péré M., Roquilly A., Riche V.P., Asehnoune K. (2023). Cost effectiveness and long-term outcomes of dexamethasone administration in major non-cardiac surgery. J. Clin. Anesth..

[33] Barden A., Phillips M., Shinde S., Corcoran T., Mori T.A. (2021). The effects of perioperative dexamethasone on eicosanoids and mediators of inflammation resolution: A sub-study of the PADDAG trial. Prostaglandins Leukot. Essent. Fatty Acids.

[34] Hopewell S., Chan A.W., Collins G.S., Hróbjartsson A., Moher D., Schulz K.F., Tunn R., Aggarwal R., Berkwits M., Berlin J.A. (2025). CONSORT 2025 statement: updated guideline for reporting randomised trials. BMJ.

[35] Santonocito C., De Loecker I., Donadello K., Moussa M.D., Markowicz S., Gullo A., Vincent J.L. (2014). C-reactive protein kinetics after major surgery. Anesth. Analg..

[36] Adamina M., Steffen T., Tarantino I., Beutner U., Schmied B.M., Warschkow R. (2015). Meta-analysis of the predictive value of C-reactive protein for infectious complications in abdominal surgery. Br. J. Surg..

[37] Stark P.A., Myles P.S., Burke J.A. (2013). Development and psychometric evaluation of a postoperative quality of recovery score: the QoR-15. Anesthesiology.

[38] Miranda F., Gonzalez F., Plana M.N., Zamora J., Quinn T.J., Seron P. (2023). Confusion Assessment Method for the Intensive Care Unit (CAM-ICU) for the diagnosis of delirium in adults in critical care settings. Cochrane Database Syst. Rev..

