# Supplementary material for: Impact of dexamethasone on postoperative inflammatory markers and recovery outcomes in elderly patients undergoing open lumbar surgery
Source: iScience. 2025 Nov 19;28(12):114133. doi: 10.1016/j.isci.2025.114133 (PMC12723133; doi:10.1016/j.isci.2025.114133)
Supplement: Document S1. Figures S1–S4 and Tables S1–S8 [file mmc1.pdf]

iScience, Volume *28*

Supplemental information

Impact of dexamethasone on postoperative inflammatory markers and recovery outcomes in elderly patients undergoing open lumbar surgery

Huiwen Zhang, Simin Liang, Hui Zhang, Fan Yang, Yonghai Zhang, Feng Wang, Yue Wen, Shaoling Ma, Zhaohui Ge, and Hanxiang Ma

Supplementary Figures and Tables

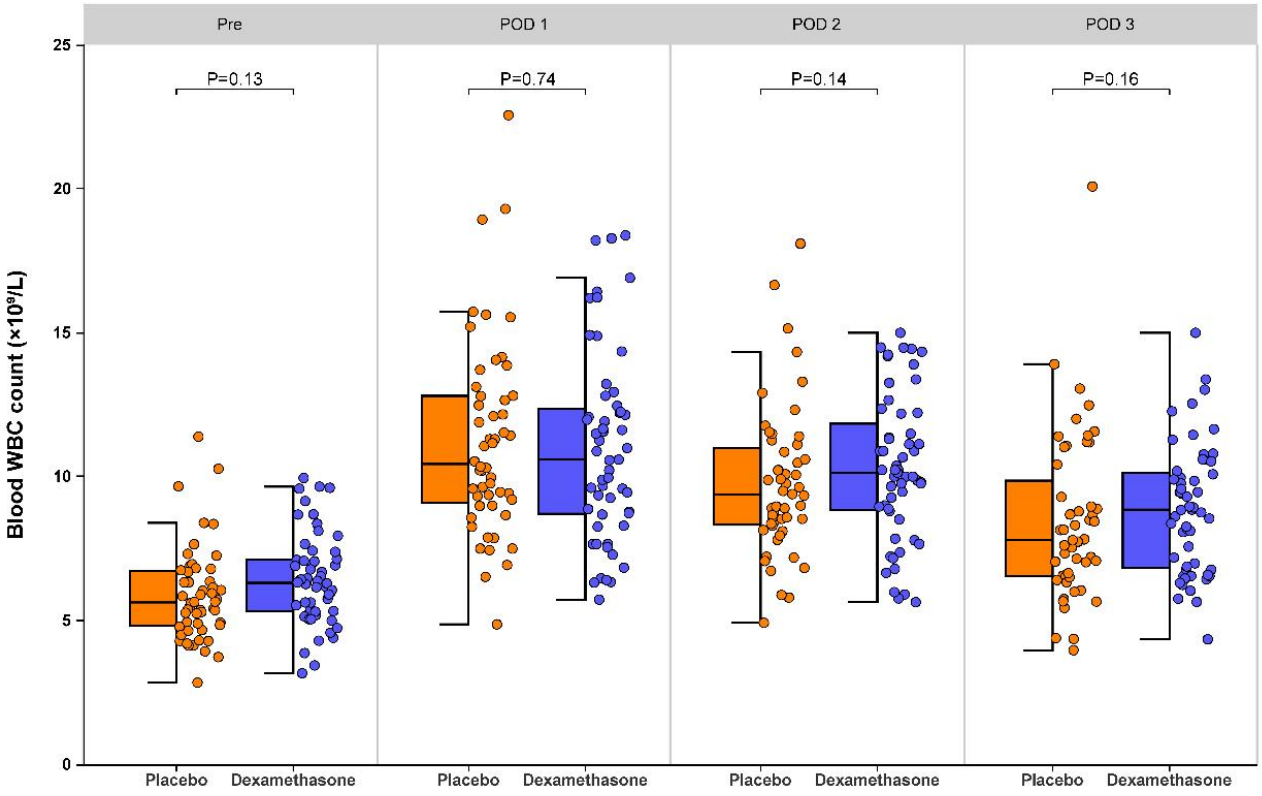

Figure S1. Blood WBC count at perioperative time.

Present the data as a combined box-and-whisker plot with an adjacent scatter plot. In the box plot on the left, the box depicts the interquartile range, the horizontal line marks the median, and the whiskers denote the minimum and maximum values. In the scatter plot on the right, each circle indicates the data-point distribution density. The Mann–Whitney U test was used.

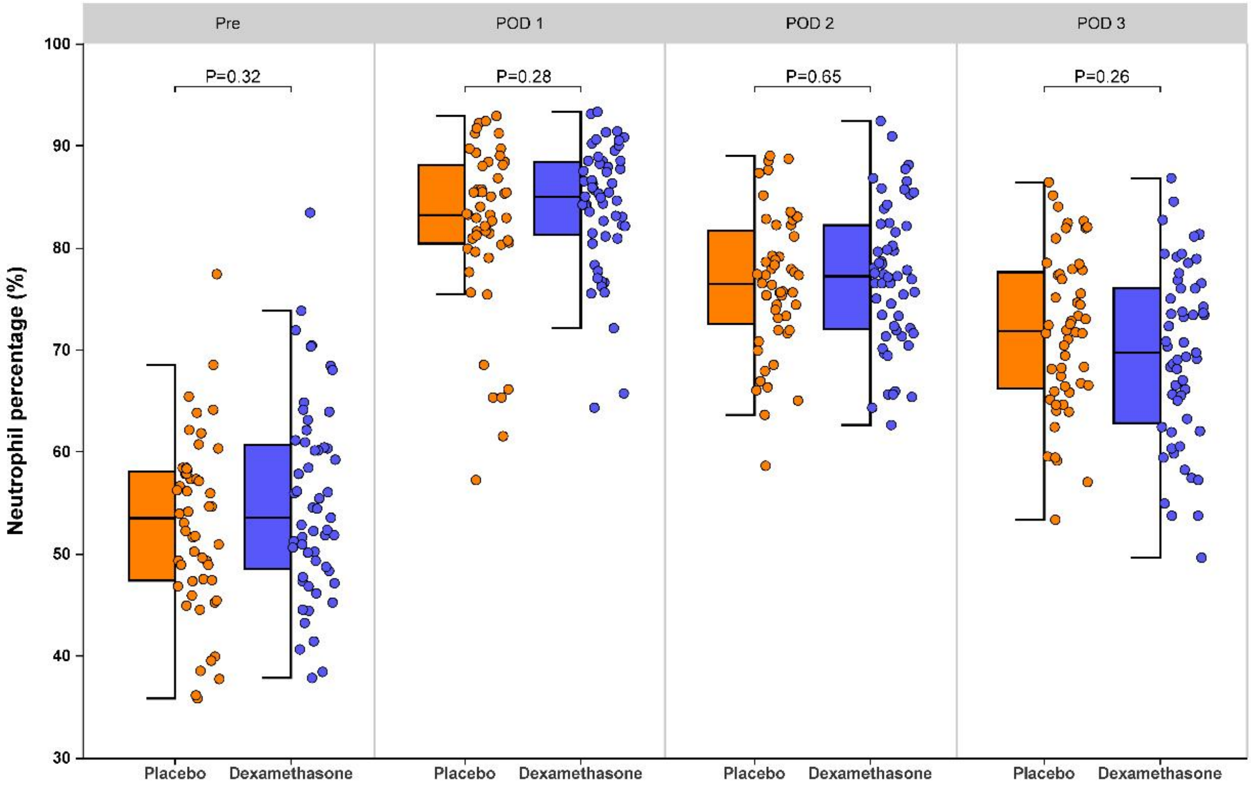

Figure S2. Blood neutrophil percentage at perioperative time.

Present the data as a combined box-and-whisker plot with an adjacent scatter plot. In the box plot on the left, the box depicts the interquartile range, the horizontal line marks the median, and the whiskers denote the minimum and maximum values. In the scatter plot on the right, each circle indicates the data-point distribution density. The Mann–Whitney U test was used.

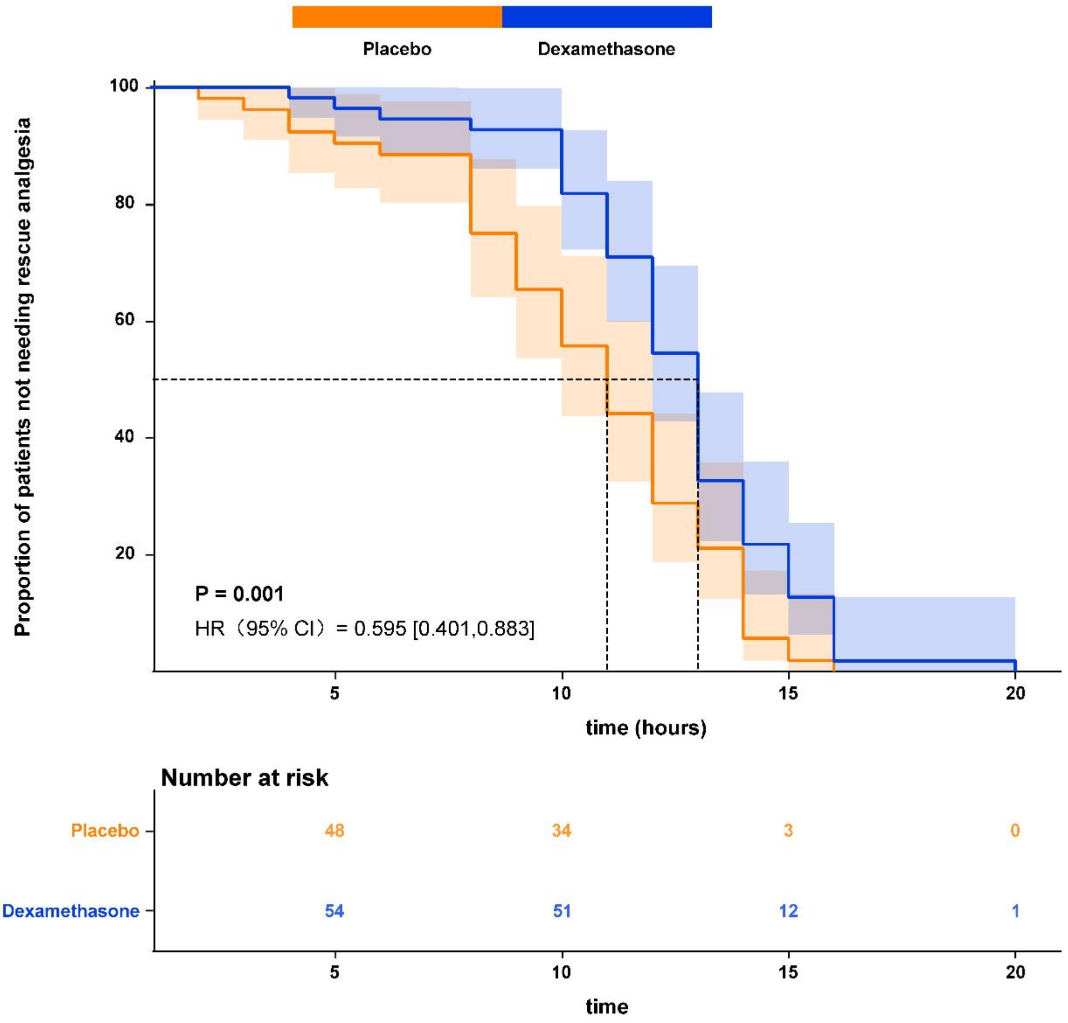

Figure S3. Kaplan-Meier survival plot representing the rescue analgesia request between the two groups.

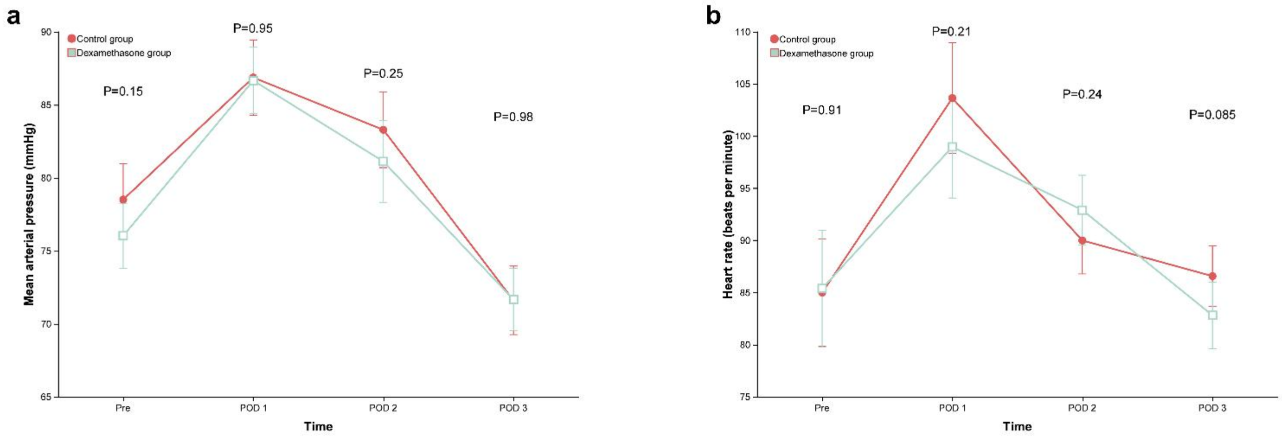

Figure S4. Non-invasive haemodynamic variables during the observational period.

Table S1 Blood IL-6 during the perioperative interval.

Time	Control group (n = 52)	Dexamethasone group (n = 55)	Median difference (95 % CI)
**Pre operative (pg/mL)**, Median (IQR) [range]	3.37 (2.40-4.31) [1.46-11.72]	3.12 (2.22-5.03) [2.00-12.52]	0.25 (-0.29 to 1.30)
**POD 1 (pg/mL)**, Median (IQR) [range]	16.35 (15.37-19.90) [10.33-42.94]	14.57 (12.24-16.50) [3.09-32.42]	1.78 (0.22 to 3.31)
**POD 2 (pg/mL)**, Median (IQR) [range]	29.32 (25.65-35.19) [19.62-77.65]	23.45 (21.63-27.05) [9.87-59.70]	5.87 (2.96 to 10.00)
**POD 3 (pg/mL)**, Median (IQR) [range]	16.82 (14.14-19.69) [3.82-50.23]	12.18 (9.94-15.44) [4.09-26.58]	4.64 (2.90 to 6.60)

Data are expressed as median (IQR) [range]. IL-6, interleukin-6; POD, postoperative day; IQR, interquartile range;

Table S2 Blood TNF-α during the perioperative interval.

Time	Control group (n = 52)	Dexamethasone group (n = 55)	Median difference (95 % CI)
**Pre operative (pg/mL)**, Median (IQR) [range]	6.83 (5.88-7.89) [2.23-10.80]	6.52 (5.69-7.76) [2.00-11.87]	0.32 (-0.01 to 1.30)
**POD 1 (pg/mL)**, Median (IQR) [range]	14.54 (12.86-17.65) [10.21-29.19]	12.15 (9.15-13.79) [2.00-21.34]	2.39 (1.16 to 4.21)
**POD 2 (pg/mL)**, Median (IQR) [range]	9.85 (8.75-10.80) [6.22-18.85]	8.22 (7.04-9.57) [2.00-13.23]	1.63 (0.67 to 2.35)
**POD 3 (pg/mL)**, Median (IQR) [range]	6.62 (5.72-7.76) [4.73-17.38]	7.01 (5.71-8.29) [2.00-11.06]	-0.38 (-0.95 to 0.50)

Data are expressed as median (IQR) [range]. TNF-α, tumour necrosis factor-α; POD, postoperative day; IQR, interquartile range;

Table S3 Blood IL-8 count during the perioperative interval.

Time	Control group (n = 52)	Dexamethasone group (n = 55)	Median difference (95 % CI)
**Pre operative (pg/mL)**, Median (IQR) [range]	43.66 (40.25-46.59) [29.88-85.41]	41.30 (36.98-45.76) [32.33-95.36]	2.37 (-0.57 to 4.97)
**POD 1 (pg/mL)**, Median (IQR) [range]	191.02 (144.77-221.84) [57.57-313.24]	155.69 (141.77-185.56) [40.85-231.54]	35.33 (15.46 to 56.01)
**POD 2 (pg/mL)**, Median (IQR) [range]	289.11 (257.22-344.67) [188.65-542.89]	245.65 (226.78-265.14) [185.43-455.69]	43.46 (21.14 to 77.42)
**POD 3 (pg/mL)**, Median (IQR) [range]	143.69 (100.58-180.55) [70.31-273.14]	110.46 (99.88-135.12) [69.78-247.65]	33.24 (7.41 to 55.05)

Data are expressed as median (IQR) [range]. IL-6, interleukin-6; POD, postoperative day; IQR, interquartile range;

Table S4 Blood WBC count during the perioperative interval.

Time	Control group (n = 52)	Dexamethasone group (n = 55)	Median difference (95 % CI)
**Pre operative (×10⁹ /L)**, Median (IQR) [range]	5.8 (4.9-6.9) [2.8-11.4]	6.3 (5.3-7.1) [3.2-9.9]	-0.5 (-1.1 to 0.1)
**POD 1 (×10⁹ /L)**, Median (IQR) [range]	10.4 (9.1-12.8) [4.9-22.6]	10.6 (8.7-12.3) [5.7-18.4]	-0.2 (-1.6 to 1.6)
**POD 2 (×10⁹ /L)**, Median (IQR) [range]	9.4 (8.3-10.9) [4.9-18.1]	10.1 (8.8-11.8) [5.6-15.0]	0.8 (-1.9 to 0.1)
**POD 3 (×10⁹ /L)**, Median (IQR) [range]	7.8 (6.5-9.6) [4.0-20.1]	8.8 (6.8-10.1) [4.3-15.0]	-1.0 (-2.0 to 0.0)

Data are expressed as median (IQR) [range]. WBC, white blood cell; POD, postoperative day; IQR, interquartile range;

Table S5 Blood neutrophil percentage during the perioperative interval.

Time	Control group (n = 52)	Dexamethasone group (n = 55)	Median difference (95 % CI)
**Pre operative (%)**, Median (IQR) [range]	53.5 (47.4-57.9) [35.8-77.4]	53.5 (48.5-60.7) [37.8-83.4]	-0.05 (-5.6 to 4.1)
**POD 1 (%)**, Median (IQR) [range]	83.2 (78.1-87.1) [56.8-94.5]	85.0 (80.7-88.8) [60.1-96.4]	-1.8 (-4.1 to 1.1)
**POD 2 (%)**, Median (IQR) [range]	76.4 (70.8-81.5) [54.9-90.3]	77.2 (72.5-83.4) [55.6-91.4]	-0.8 (-2.9 to 1.8)
**POD 3 (%)**, Median (IQR) [range]	71.8 (66.3-77.5) [53.3-86.4]	69.7 (62.8-76.0) [49.6-86.8]	2.1 (-2.5 to 5.5)

Data are expressed as median (IQR) [range]. POD, postoperative day; IQR, interquartile range;

Table S6 Blood glucose during the perioperative interval.

Time	Control group (n = 52)	Dexamethasone group (n = 55)	Median difference (95 % CI)
**Pre operative**, Median (IQR) [range]	4.9 (4.6-5.5) [4.2-7.8]	4.9 (4.5-5.6) [3.9-11.1]	-0.1 (-0.3 to 0.4)
**POD 1**, Median (IQR) [range]	5.8 (5.4-6.3) [4.0-8.8]	6.4 (5.9-7.4) [4.5-12.3]	-0.6 (-0.9 to -0.2)
**POD 2**, Median (IQR) [range]	5.4 (4.8-5.6) [4.4-7.4]	5.5 (5.3-6.4) [4.3-9.9]	-0.1 (-0.5 to -0.1)
**POD 3**, Median (IQR) [range]	5.0 (4.6-5.2) [3.9-7.3]	5.2 (4.7-5.5) [4.1-8.7]	-0.3 (-0.7 to 0.1)

Data are expressed as median (IQR) [range]. POD, postoperative day; IQR, interquartile range;

Table S7 Pain score during the perioperative interval.

Time	Control group (n = 52)	Dexamethasone group (n = 55)	Median difference (95 % CI)
**Pre operative**, Median (IQR) [range]	3 (2-3) [0-6]	3 (2-3) [0-5]	0 (0 to 0)
**POD 1**, Median (IQR) [range]	4 (4-5) [3-6]	3 (2-4) [0-6]	1 (0 to 2)
**POD 2**, Median (IQR) [range]	4 (3-5) [0-6]	4 (3-5) [0-5]	0 (0 to 2)
**POD 3**, Median (IQR) [range]	2 (2-4) [0-5]	3 (2-4) [0-5]	-1 (-2 to 0)

Data are expressed as median (IQR) [range]. POD, postoperative day; IQR, interquartile range;

Table S8 QoR-15 score during the perioperative interval.

Time	Control group (n = 52)	Dexamethasone group (n = 55)	Median difference (95 % CI)
**Pre operative**, Median (IQR) [range]	145 (140-147) [138-150]	143 (141-145) [138-149]	2 (-1 to 3)
**POD 1**, Median (IQR) [range]	118 (115-128) [105-130]	132 (120-138) [112-145]	-14 (-17 to -6)
**POD 2**, Median (IQR) [range]	124 (119-130) [116-139]	138 (126-138) [122-148]	-14 (-16 to -5)
**POD 3**, Median (IQR) [range]	131 (123-138) [119-142]	139 (130-144) [122-147]	-8 (-12 to -1)

Data are expressed as median (IQR) [range]. QoR, Quality of recovery; POD, postoperative day; IQR, interquartile range;
